# Do positive psychosocial factors contribute to the prediction of coronary artery disease? A UK Biobank–based machine learning approach

**DOI:** 10.1093/eurjpc/zwae237

**Published:** 2024-07-26

**Authors:** René Hefti, Souad Guemghar, Edouard Battegay, Christian Mueller, Harold G Koenig, Rainer Schaefert, Gunther Meinlschmidt

**Affiliations:** Department of Psychosomatic Medicine, University Hospital Basel and University of Basel, Hebelstrasse 2, CH-4031 Basel, Switzerland; Department of Psychosomatic Medicine, University Hospital Basel and University of Basel, Hebelstrasse 2, CH-4031 Basel, Switzerland; Department of Psychosomatic Medicine, University Hospital Basel and University of Basel, Hebelstrasse 2, CH-4031 Basel, Switzerland; International Center for Multimorbidity and Complexity in Medicine (ICMC), University of Zurich, Rämistrasse 71, CH-8006 Zurich, Switzerland; Merian Iselin Klinik, Föhrenstrasse 2, CH-4054 Basel, Switzerland; Cardiovascular Research Institute, University Hospital Basel, Petersgraben 4, CH-4031 Basel, Switzerland; Department of Medicine and Psychiatry, Duke University Medical Center, 40 Duke Medicine Cir., Durham, NC 27710, USA; Department of Psychosomatic Medicine, University Hospital Basel and University of Basel, Hebelstrasse 2, CH-4031 Basel, Switzerland; Department of Psychosomatic Medicine, University Hospital Basel and University of Basel, Hebelstrasse 2, CH-4031 Basel, Switzerland; Department of Digital and Blended Psychosomatics and Psychotherapy, Psychosomatic Medicine, University Hospital Basel and University of Basel, Hebelstrasse 2, CH-4031 Basel, Switzerland; Department of Psychology, Clinical Psychology and Psychotherapy—Methods and Approaches, Trier University, Universitaetsring 15, D-54296 Trier, Germany; Department of Clinical Psychology and Cognitive Behavioural Therapy, International Psychoanalytic University (IPU) Berlin, Stromstrasse 3b, D-10555 Berlin, Germany

**Keywords:** Cardiovascular disease, Positive psychosocial factors, Disease prediction, Artificial intelligence, Preventive cardiology

## Abstract

**Aims:**

Most prediction models for coronary artery disease (CAD) compile biomedical and behavioural risk factors using linear multivariate models. This study explores the potential of integrating positive psychosocial factors (PPFs), including happiness, satisfaction with life, and social support, into conventional and machine learning–based CAD‐prediction models.

**Methods and results:**

We included UK Biobank (UKB) participants without CAD at baseline. First, we estimated associations of individual PPFs with subsequent acute myocardial infarction (AMI) and chronic ischaemic heart disease (CIHD) using logistic regression. Then, we compared the performances of logistic regression and eXtreme Gradient Boosting (XGBoost) prediction models when adding PPFs as predictors to the Framingham Risk Score (FRS). Based on a sample size between 160 226 and 441 419 of UKB participants, happiness, satisfaction with health and life, and participation in social activities were linked to lower AMI and CIHD risk (all *P*-for-trend ≤ 0.04), while social support was not. In a validation sample, adding PPFs to the FRS using logistic regression and XGBoost prediction models improved neither AMI [area under the receiver operating characteristic curve (AUC) change: 0.02 and 0.90%, respectively] nor CIHD (AUC change: −1.10 and −0.88%, respectively) prediction.

**Conclusion:**

Positive psychosocial factors were individually linked to CAD risk, in line with previous studies, and as reflected by the new European Society of Cardiology guidelines on cardiovascular disease prevention. However, including available PPFs in CAD‐prediction models did not improve prediction compared with the FRS alone. Future studies should explore whether PPFs may act as CAD-risk modifiers, especially if the individual’s risk is close to a decision threshold.

## Graphical Abstract

**Graphical Abstract:**
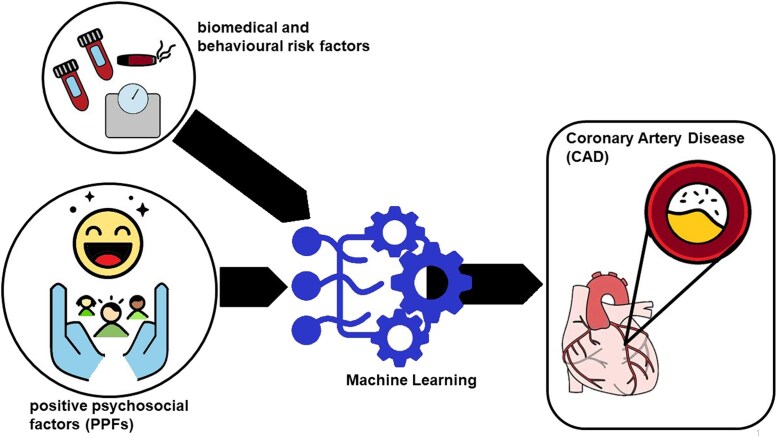

**See the editorial comment for this article ‘Positive psychology goes cardiology: what we have learned and what's next’, by R. von Känel, https://doi.org/10.1093/eurjpc/zwae228.**

## Introduction

Coronary artery disease (CAD) is the most common heart disease and the leading cause of death globally (‘CardioPulse’). Although common, it remains preventable. Risk factor identification, preventive strategies, and advancements in medical treatment have significantly reduced CAD mortality.^1,2^ Still, given the individual, societal, and financial burden of CAD, there are ongoing efforts to further reduce its incidence.^3^

Approaches to prevent cardiovascular events and mortality have focused predominantly on biomedical and behavioural risk factors,^3–5^ using multivariate approaches that assume a linear relationship between predictors and outcome.^6^ This is the case for the Framingham Risk Score (FRS), recommended by the American College of Cardiology/American Heart Association,^7^ and the Systematic Coronary Risk Evaluation (SCORE), recommended by the European Society of Cardiology (ESC) clinical practice guidelines.^8^

During the last decade, psychosocial risk factors including depression, anxiety, hostility, work-related stress, vital exhaustion, low socio-economic status, social isolation, Type D personality, and post-traumatic stress disorder have been linked to cardiovascular disease progression.^9–12^ As a result, psychosocial factors have primarily been included in the ESC Guidelines on cardiovascular disease prevention.^8^

Schnohr *et al*.^13^ compared psychosocial factors against typical biomedical risk factors using different predictive models and data from the Copenhagen City Heart Study. Vital exhaustion was the strongest predictor, stronger than systolic blood pressure, and significantly improved risk prediction based on the SCORE model.

However, only limited research has investigated the associations between CAD and so-called positive psychosocial factors (PPFs) such as subjective well-being, happiness, optimism, purpose in life, spirituality and perceived social support, and their potentially protective effects.^14–23^

Therefore, we aimed to explore the association of PPFs with CAD in a large prospective cohort study and to assess its predictive power compared with a well-established risk score. We decided on the office-based non-laboratory version of the FRS incorporating body mass index instead of total and HDL cholesterol, showing a good performance in Framingham study participants^6^ and facilitating routine and remote risk assessment in preventive cardiology.^24,25^ Our study sought to (i) estimate individual associations of PPF happiness, satisfaction with health and life, social support, and social activities with two CAD endpoints, namely acute myocardial infarction (AMI) and chronic ischaemic heart disease (CIHD), and (ii) evaluate the potential of PPFs to improve multivariate and machine learning (ML)-based predictions of AMI and CIHD, by comparing the prediction performance between these methods. Analyses were based on the UK Biobank (UKB),^26^ a large prospective cohort study conducted in the UK.

## Methods

### Study design and population

This observational study is based on data from the UKB, a population-based national cohort of 502 393 UK residents recruited between 2006 and 2010, and assessed at 22 assessment centres in England, Scotland, and Wales. Data from the initial assessment, primary care records, in-patient records, and death registers were all used for this study. The UKB also includes follow-up assessments, follow-up online questionnaires, and cancer registry data. All UKB participants provided written informed consent on a touchscreen at baseline assessment. The UKB received ethical approval from the NorthWest Multi-Centre Research Ethics Committee (REC reference: 11/NW/03820). The UKB approved our use of the data for this study under application number 85966.

We excluded participants with a history of CAD at baseline assessment. That is, we excluded participants who had angina pectoris, AMI, subsequent myocardial infarction, complications following AMI, CIHD, and other acute ischaemic heart disease, as indicated by International Statistical Classification of Diseases and Related Health Problems, 10th Revision (ICD-10) Codes I20–I25, and reported by at least one of primary care, hospital admissions, death registry data, or self-report.

### Primary outcomes

The primary outcomes of this study were the diagnosis of AMI or CIHD, indicated by ICD-10 Codes I21 and I25, respectively. A participant was considered to have an AMI or CIHD diagnosis if they had a valid date on which AMI (I21) or CIHD (I25) was first reported.

### Predictors

The PPFs included in the baseline assessment were: general happiness (‘In general, how happy are you?’); satisfaction with health (‘In general, how satisfied are you with your health?’); satisfaction with life (‘In general, how satisfied are you with your family relationships?’; ‘In general, how satisfied are you with your friendships?’; ‘In general, how satisfied are you with your financial situation?’; ‘In general, how satisfied are you with the work that you do?’) and social support (‘How often do you visit friends or family or have them visit you?; Do you attend leisure or social activities?; How often are you able to confide in someone close to you?)’. All PPFs are assessed as single items; therefore, we treated them as categorical variables, as detailed in the Supplementary material online, *Table S1*.

As the UKB did not use standardized scales for happiness, satisfaction with health and life, or social support, neither at baseline nor in subsequent assessments such as the Mental Health Questionnaire, the PPF items were assigned to the psychological constructs in line with previous UKB publications.^27,28^

### Associations

We conducted logistic regression analysis for each PPF to estimate the association of available PPFs with subsequent AMI and CIHD. We treated every response category as a separate categorical predictor. Subsequently, we conducted *P*-for-trend analyses for each individual PPF to test for a dose–response relationship, by ordering the PPF response categories according to intensity. For the *P*-for-trend analyses, we excluded the dichotomous leisure and social activities variable and participants with non-quantifiable response categories—do not know, prefer not to answer, no friends/family outside household, and I am not employed.

### Prediction models

We developed logistic regression and XGBoost models to predict two outcomes: AMI and CIHD. Given that ML is not yet fully established in cardiovascular medicine, we chose to complement the XGBoost model with logistic regression to provide a more robust comparative analysis. Logistic regression is frequently used as a baseline model due to its simplicity of implementation and interpretability. More sophisticated algorithms (tree-based or neural networks) have to outperform it to be useful. Indeed, if ML approaches do not demonstrate superior performance over logistic regression, their use may not be warranted due to the increased complexity of implementation and interpretability.

Nevertheless, the potential of ML techniques to uncover non-linear relationships and interactions between variables, and their ability to handle complex data patterns, warrant further exploration. Investing in ML approaches may lead to advancements in predictive accuracy and deeper insights into cardiovascular outcomes, even if immediate gains are not evident. Prior to training the models, we split the data set into training (80%) and test (20%) data sets, stratified on the cardiovascular outcomes AMI and CIHD.

The office-based version of the FRS constituted our baseline model. Supplementary material online, *Table S2* details the variables we used to replicate the FRS in the UKB. We calculated an FRS for each participant using the formulas for males and females published in the original article.^6^ For this baseline model, we excluded 33 229 participants who had missing values in any of the variables necessary to calculate the FRS. In the logistic regression models, we used individual PPFs as predictors, adjusting them for the participants’ sex, age at recruitment, Townsend deprivation index, non-lab FRS, and depression as measured by the ‘Frequency of depressed mood in last 2 weeks’ item.

### Statistical analysis

We performed all calculations at sciCORE,^29^ the scientific computing centre at the University of Basel, using R version 4.1.2. In addition to base R, we used several packages, including the tidyverse^30^ collection of packages for data manipulation and visualization, and the tidymodels^31^ framework to build prediction models and measure their performance (Supplementary material online, *Table S3*). We estimated feature relevance within the models, using variable importance measurements, in the form of scores of the model’s predictors.

We addressed the issue of missing data by conducting completer analyses for all logistic regression models, keeping only features with fewer than 70% missing data. Missing categorical variables were assigned a new ‘unknown’ category in XGBoost models. Since XGBoost models accept only numeric variables, we encoded all categorical variables into dummy variables. Furthermore, we tuned XGBoost’s hyperparameters using five-fold crossvalidation, a size 30 space-filling parameter grid, and racing methods^32^ to speed up computations. Given the low incidence of AMI (2.2%) and CIHD (6.3%) in our sample, we assessed the models using the area under the receiver operating characteristic curve (AUC). The held out 20% test set was used for performance evaluation, and the confidence interval (CI) of the AUC was computed with 2000 stratified bootstrap replicates. We provided AUC estimates with 95% CI of the models on the testing set, in accordance with two-tailed *P*-values with a statistical significance level of 0.05.

For reporting, we followed the Transparent Reporting of a Multivariable Prediction Model for Individual Prognosis or Diagnosis (TRIPOD) guidelines, which are detailed in the Supplementary material online, *Table S4*.

## Results

We excluded 27 214 UKB participants with CAD at baseline assessment. Of the 475 175 included participants, 10 650 were diagnosed with AMI, and 30 141 were diagnosed with CIHD following baseline assessment. *Table 1* shows detailed sociodemographic characteristics of the participants included in the study. In calculating the associations of PPFs with AMI/CIHD sample size, between 160 226 and 441 419 UKB participants were found eligible depending on the availability of individual PPFs (see *Table 2*).

**Table 1:** Demographic characteristics of the UK Biobank participants included in this study

Variable	*n* = 475 175^a^
Acute myocardial infarction^b^	10 650 (2.2%)
Chronic ischaemic heart disease^c^	30 141 (6.3%)
Sex	
Female	264 773 (56%)
Male	210 425 (44%)
Age at recruitment (in years)^d^	
Median (IQR)	57 (50–63)
Townsend deprivation index^e^	
Median (IQR)	−2.17 (−3.66, 0.48)
Ethnic background^f^	
White	447 174 (95%)
South Asian	8990 (1.9%)
Other	8698 (1.8%)
Black	7728 (1.6%)
Body mass index^g^ (kg/m^2^)	
Median (IQR)	26.6 (24.1–29.8)
Alcohol drinker status^h^	
Current	436 650 (92%)
Never	20 675 (4.4%)
Previous	16 318 (3.4%)
Prefer not to answer	692 (0.1%)
Smoking status^i^	
Never	263 129 (55%)
Previous	159 803 (34%)
Current	49 561 (10%)
Prefer not to answer	1846 (0.4%)
Framingham Risk Score^j^	
Median (IQR)	0.13 (0.07–0.22)
Overall health rating^k^	
Excellent	80 883 (17%)
Good	278 705 (59%)
Fair	94 482 (20%)
Poor	17 949 (3.8%)
Do not know	1959 (0.4%)
Prefer not to answer	338 (<0.1%)

IQR, interquartile range.

^a^ *n* (%). Excluded 27 214 participants diagnosed with coronary artery disease before baseline assessment.

^b^First diagnosis of acute myocardial infarction after baseline assessment between 7 July 2006 and 12 November 2021.

^c^First diagnosis of chronic ischaemic heart disease after baseline assessment between 6 July 2006 and 18 October 2021.

^d^One participant had no information on his age at recruitment.

^e^The Townsend index is a measure of material deprivation (poverty) within a population incorporating variables like unemployment, car-, and homeownership. Five hundred and eighty-eight participants had no information on their Townsend deprivation index.

^f^Category ‘White’ includes British, Irish, and any other white background. ‘Black’ includes Caribbean, African, and any other black background. ‘South Asian’ includes Indian, Pakistani, Bangladeshi, and any other South Asian background. ‘Other’ includes mixed, Chinese, or other ethnicities. About 2585 had no information on their ethnic background.

^g^About 2807 participants had no information on their body mass index.

^h^About 840 participants had no information on their alcohol drinking status.

^i^About 836 participants had no information on their smoking status.

^j^We could not calculate the Framingham Risk Score of 33 229 participants because they lacked information on one of the core variables necessary to calculate it.

^k^About 859 participants had no health rating.

**Table 2:** Adjusted associations of positive psychosocial factors with coronary artery disease including *P*-for-trend analyses

Variable	AMI			CIHD		
	OR	95% CI	*P*-value	OR	95% CI	*P*-value
General happiness						
In general how happy are you (*n* = 160 226)						
Very or extremely unhappy	Reference category			Reference category		
Moderately unhappy	0.6	0.43, 0.84	0.003	0.74	0.60, 0.91	0.004
Moderately happy	0.63	0.48, 0.86	0.002	0.67	0.56, 0.81	<0.001
Very happy	0.62	0.47, 0.85	0.002	0.63	0.53, 0.77	<0.001
Extremely happy	0.67	0.49, 0.94	0.017	0.63	0.52, 0.78	<0.001
Do not know	0.82	0.47, 1.36	0.4	0.83	0.60, 1.14	0.3
Prefer not to answer	0.53	0.18, 1.27	0.2	1.08	0.67, 1.69	0.7
*P* for trend	0.79	0.63, 0.99	0.039	0.71	0.62, 0.82	<0.001
Satisfaction with health						
In general how satisfied are you with your health (*n* = 160 226)						
Extremely unhappy	Reference category			Reference category		
Very unhappy	0.99	0.71, 1.41	>0.9	0.81	0.68, 0.98	0.028
Moderately unhappy	0.79	0.59, 1.10	0.15	0.59	0.50, 0.70	<0.001
Moderately happy	0.72	0.54, 0.99	0.035	0.46	0.39, 0.54	<0.001
Very happy	0.6	0.45, 0.83	0.001	0.33	0.29, 0.39	<0.001
Extremely happy	0.6	0.43, 0.85	0.004	0.33	0.27, 0.40	<0.001
Do not know	1.06	0.63, 1.74	0.8	0.53	0.38, 0.71	<0.001
Prefer not to answer	0.77	0.26, 1.85	0.6	0.77	0.46, 1.23	0.3
*P* for trend	0.61	0.49, 0.77	<0.001	0.36	0.32, 0.41	<0.001
Satisfaction with life						
Family relationship satisfaction (*n* = 160 226)						
Very or extremely unhappy	Reference category			Reference category		
Moderately unhappy	0.66	0.50, 0.87	0.003	0.79	0.67, 0.93	0.005
Moderately happy	0.71	0.57, 0.89	0.002	0.75	0.65, 0.86	<0.001
Very happy	0.71	0.57, 0.90	0.003	0.72	0.63, 0.83	<0.001
Extremely happy	0.69	0.55, 0.88	0.002	0.74	0.64, 0.86	<0.001
Do not know	0.61	0.38, 0.95	0.033	0.79	0.61, 1.01	0.061
Prefer not to answer	0.91	0.54, 1.45	0.7	0.95	0.70, 1.26	0.7
*P* for trend	0.82	0.70, 0.96	0.013	0.8	0.73, 0.88	<0.001
Friendships satisfaction (*n* = 160 226)						
Very or extremely unhappy	Reference category			Reference category		
Moderately unhappy	0.69	0.45, 1.07	0.088	0.6	0.47, 0.78	<0.001
Moderately happy	0.73	0.51, 1.08	0.1	0.62	0.50, 0.77	<0.001
Very happy	0.76	0.53, 1.13	0.2	0.61	0.50, 0.76	<0.001
Extremely happy	0.8	0.55, 1.20	0.3	0.63	0.51, 0.79	<0.001
Do not know	0.85	0.53, 1.38	0.5	0.61	0.46, 0.80	<0.001
Prefer not to answer	0.42	0.16, 0.92	0.042	0.72	0.48, 1.05	0.093
*P* for trend	0.89	0.70, 1.17	0.4	0.74	0.64, 0.87	<0.001
Financial situation satisfaction (*n* = 160 226)						
Extremely unhappy	Reference category			Reference category		
Very unhappy	0.8	0.60, 1.07	0.13	0.75	0.63, 0.89	<0.001
Moderately unhappy	0.81	0.63, 1.05	0.11	0.73	0.63, 0.85	<0.001
Moderately happy	0.73	0.58, 0.93	0.008	0.62	0.54, 0.72	<0.001
Very happy	0.62	0.49, 0.80	<0.001	0.56	0.49, 0.65	<0.001
Extremely happy	0.65	0.49, 0.85	0.002	0.56	0.48, 0.65	<0.001
Do not know	0.82	0.46, 1.37	0.5	0.82	0.60, 1.10	0.2
Prefer not to answer	0.8	0.45, 1.35	0.4	0.78	0.57, 1.06	0.12
*P* for trend	0.69	0.58, 0.84	<0.001	0.63	0.56, 0.70	<0.001
Work or job satisfaction (*n* = 160 226)						
Very or extremely unhappy	Reference category			Reference category		
Moderately unhappy	0.94	0.69, 1.30	0.7	0.93	0.77, 1.13	0.5
Moderately happy	0.95	0.73, 1.27	0.7	0.91	0.78, 1.08	0.3
Very happy	0.91	0.70, 1.22	0.5	0.91	0.77, 1.08	0.3
Extremely happy	1.04	0.77, 1.41	0.8	0.87	0.73, 1.05	0.15
I am not employed	0.96	0.73, 1.28	0.8	0.93	0.79, 1.10	0.4
Do not know	1.02	0.56, 1.76	>0.9	0.96	0.68, 1.34	0.8
Prefer not to answer	0.86	0.41, 1.65	0.7	1.27	0.88, 1.80	0.2
*P* for trend	0.98	0.80, 1.21	0.9	0.86	0.76, 0.97	0.016
Social support						
Frequency of friend or family visits (*n* = 440 452)						
Never or almost never	Reference category			Reference category		
Once every few months	0.84	0.72, 0.99	0.04	0.9	0.81, 0.99	0.037
About once a month	0.81	0.70, 0.95	0.008	0.85	0.77, 0.93	<0.001
About once a week	0.89	0.78, 1.04	0.13	0.91	0.83, 0.99	0.033
2–4 times a week	0.93	0.80, 1.07	0.3	0.91	0.83, 1.00	0.053
Almost daily	0.96	0.83, 1.12	0.6	1.01	0.92, 1.11	0.8
No friends/family outside household	0.99	0.67, 1.41	>0.9	1.23	0.98, 1.53	0.063
Do not know	1.22	0.88, 1.68	0.2	1.08	0.87, 1.33	0.5
Prefer not to answer	0.87	0.57, 1.27	0.5	0.87	0.68, 1.11	0.3
*P* for trend	1.02	0.93, 1.13	0.7	1.02	0.96, 1.09	0.5
Leisure or social activities (*n* = 439 897)	0.92	0.88, 0.96	<0.001	0.94	0.91, 0.96	<0.001
Able to confide (*n* = 441 419)						
Never or almost never	Reference category			Reference category		
Once every few months	0.9	0.81, 0.99	0.033	0.95	0.89, 1.01	0.11
About once a month	0.94	0.85, 1.04	0.2	0.92	0.86, 0.98	0.008
About once a week	0.92	0.85, 1.00	0.043	0.94	0.89, 0.98	0.009
2–4 times a week	0.94	0.86, 1.02	0.13	0.94	0.89, 0.99	0.013
Almost daily	0.93	0.88, 0.99	0.014	0.95	0.92, 0.98	0.004
Do not know	0.99	0.88, 1.12	>0.9	0.99	0.92, 1.07	0.8
Prefer not to answer	1.08	0.83, 1.38	0.6	1.06	0.90, 1.24	0.5
*P* for trend	0.97	0.92, 1.03	0.3	0.97	0.93, 1.00	0.041

Adjusted for participants’ sex, age at recruitment, and Townsend deprivation index, Framingham Risk Score and depression as measured by the ‘Frequency of depressed mood in last 2 weeks’ item.

AMI, acute myocardial infarction; CI, confidence interval; CIHD, chronic ischaemic heart disease; OR, odds ratio.

### Associations between positive psychosocial factors and coronary artery disease

The adjusted associations of PPFs with AMI and CIHD are shown in *Table 2*. These associations were calculated using logistic regression on the whole sample as summarized in *Table 1*. The lowest response categories served as reference categories. If these categories contained fewer than 1000 participants, they were combined with the next higher category.

*P*-for-trend estimates suggest dose–response associations. Higher levels of general happiness, satisfaction with health and life (including family relationships, finances), and participation in social activities were associated with reduced risks of AMI and CIHD. In contrast, friendship satisfaction was only associated with a reduced risk of CIHD. Social support, as measured by the frequency of friends and family visits and the ability to confide, did not demonstrate significant associations with cardiovascular endpoints.

**Figure 1:**
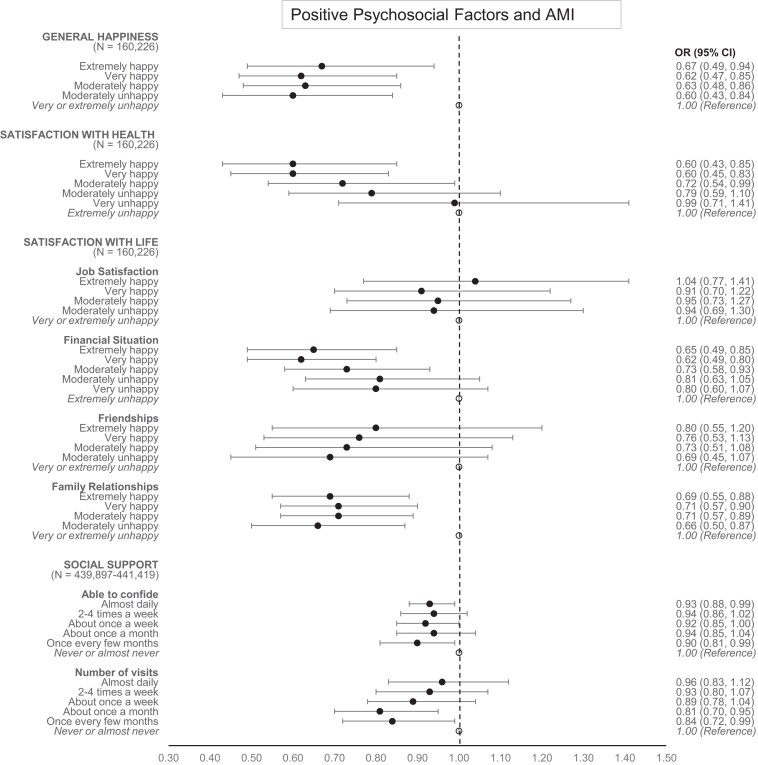
Adjusted associations of positive psychosocial factors with acute myocardial infarction: odds ratios and 95% confidence intervals of all response categories. Adjusted for the participants’ sex, age at recruitment, Townsend deprivation index, Framingham Risk Score, and depression as measured by the ‘Frequency of depressed mood in last 2 weeks’ item.

**Figure 2:**
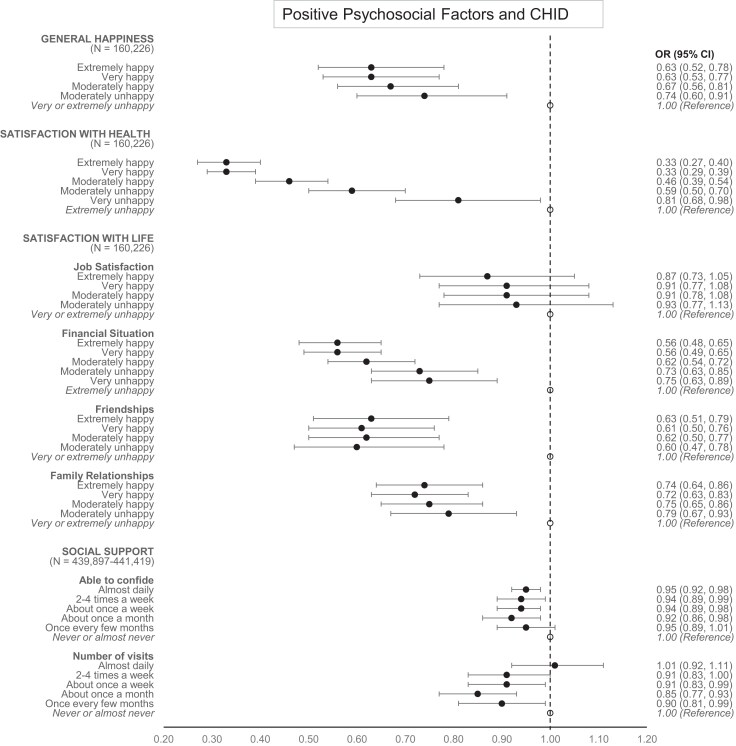
Adjusted associations of positive psychosocial factors with chronic ischaemic heart disease: odds ratios and 95% confidence intervals of all response categories. Adjusted for the participants’ sex, age at recruitment, Townsend deprivation index, Framingham Risk Score, and depression as measured by the ‘Frequency of depressed mood in last 2 weeks’ item.

*Figures 1* and *2* illustrate the associations between PPFs and AMI/CIHD using forest plots. The lowest response categories served as reference categories (see *Table 2*). *AMI results*: General happiness and family relationships indicated a similar odds ratio across all levels from ‘extremely happy’ to ‘moderately unhappy’, with CIs below 1.0. This suggests that even moderate unhappiness can significantly reduce the risk of AMI. Satisfaction with health followed a linear trend, with the lowest odds ratios found among extremely happy participants. Satisfaction with job and friendships did not demonstrate notable benefits in terms of AMI risk reduction. Satisfaction with the financial situation showed again a trend where the lowest odds ratios were seen in extremely happy individuals. In contrast, social support measures did not provide significant benefits for reducing AMI risk. *CIHD results*: The findings were largely similar to those for AMI but with generally lower odds ratios. Satisfaction with friendships demonstrated a clear benefit in reducing the risk of CIHD, while the ability to confide also appeared to slightly decrease the risk of CIHD.

The forest plots in *Figures 1* and *2* offer a more detailed view of the associations between PPFs and cardiovascular outcomes, highlighting the varying degrees of associations across different PPFs.

### Positive psychosocial factors and coronary artery disease prediction

To evaluate the predictive power of PPFs, we compared the performance of logistic regression and XGBoost models for AMI and CIHD, using the FRS as a reference model. Including PPFs in the logistic regression and XGBoost models did not improve total prediction compared with the FRS (*Table 3*).

**Table 3:** Prediction models for acute myocardial infarction and chronic ischaemic heart disease including positive psychosocial factors

	AMI			CIHD		
	AUC	95% CI	Change (%)	AUC	95% CI	Change (%)
Logistic regression^a^						
FRS^b^	0.713	0.702–0.724	Baseline	0.738	0.732–0.744	Baseline
FRS + PPF^c^	0.713	0.692–0.733	0.02	0.727	0.716–0.738	−1.10
XGBoost						
FRS	0.721	0.711–0.730	0.79	0.725	0.718–0.731	−1.31
FRS + PPF	0.722	0.712–0.732	0.90	0.729	0.723–0.735	−0.88

AMI, acute myocardial infarction; AUC, area under the receiver operating characteristic curve; CI, confidence interval; CIHD, chronic ischaemic heart disease; FRS, Framingham Risk Score calculated using variables in supplementary material online, *Table S2*; PPF, positive psychosocial factor; XGBoost, eXtreme Gradient Boosting.

^a^We removed participants with missing information in any of the predictors and retained variables with fewer than 70% missing data.

^b^About 33 229 participants were removed from analysis.

^c^About 315 352 participants were removed from analysis.

## Discussion

To our knowledge, this is the first study to investigate the potential contribution of PPFs to the prediction of CAD using UKB data, a prospective population-based national cohort of 502 393 UK residents. Most PPFs were individually linked to reduced risk of the cardiovascular endpoints AMI and CIHD. Yet, adding PPFs to the FRS did not improve prediction of AMI and CIHD, neither in logistic regression nor in XGBoost models.

### Associations between positive psychosocial factors and coronary artery disease

The estimated associations between PPFs and CAD are in line with previous findings.^12–14,16,17,20,23^ Similarly to the Swedish CArdioPulmonary bioImage Study (SCAPIS), which analysed cross-sectional data on life satisfaction and coronary atherosclerosis of 6251 participants,^33^ we identified a negative association between life satisfaction and CAD risk. In the SCAPIS cohort, higher levels of life satisfaction, a component of psychological well-being, were associated with less coronary artery calcification. For satisfaction with health, we observed a reduced risk for AMI and CIHD. The Canadian Nova Scotia Health Survey, a population-based prospective study comprising 1739 adults, examined the association of positive affect (assessed via structured interviews) with acute non-fatal or fatal ischaemic heart disease events.^16^ It concluded that positive affect was associated with lower CAD risk. In our study, general happiness was associated with lower risks of AMI and CIHD, while the Whitehall II prospective cohort study, based on 10 308 civil servants aged 35–55 years, found no evidence for an association of happiness with fatal and non-fatal CAD.^34^ These potentially disparate findings may be the result of differences in type and assessment of positive affect, cultural differences, as well as outcome measures (non-fatal or fatal events, CIHD).

Social support showed a weak association with reduced risk for CIHD and an even weaker one for AMI in the present study. This differs from the findings of the SCAPIS pilot study,^35^ which reported a significant relationship between low social support and cardiovascular risk factors, high levels of inflammatory markers, and coronary artery calcification in women, but not in men. This might be explained by the single-item scales used in the baseline assessment for social support in the UK cohort. Several studies found that low social support is a potential psychosocial risk factor for CAD.^12,36^ Also a recent meta-analysis, investigating the association between loneliness or social isolation and incident coronary heart disease, indicated that low social support was associated with a 29% increase in CAD.^37^

### Positive psychosocial factors and the prediction of coronary artery disease

We hypothesized that PPFs (happiness, satisfaction with life and health, and social support) would improve prediction of AMI and CIHD. However, despite multiple individual associations between PPFs and CAD, we did not find evidence for PPFs improving CAD prediction, using the FRS as a reference. One possible reason for this lack of incremental predictive power may be due to the limited range of PPFs assessed in the UKB. Positive psychosocial factors with the strongest association to CAD, such as well-being, optimism, purpose in life, and spirituality,^15,18,19,21,22^ were not available. The additional mental health survey conducted in 2016–17 as part of the UKB collected in-depth psychosocial data, including meaning and purpose. However, we could not use these data for our analyses, as this would have substantially reduced our sample size by excluding all CAD cases before 2016–17. Future large-scale and follow-up studies with more detailed information on PPFs are highly recommended.

### Strengths and limitations

One key strength of the present study is the large sample and prospective design, which includes over 475 000 UK residents with an average follow-up of 12 years. In addition, the UKB includes data from primary care records, in-patient records, and data from death registers, all of which are updated regularly, ensuring a high degree of follow-up coverage. Participants who move outside the UK are the only ones lost to follow-up. Another significant strength is the use of ML techniques, which enabled the identification of non-linear associations between PPFs and the outcomes.

An important limitation of the study is the number and quality of PPFs assessed at baseline. Positive psychosocial factors were assessed using single questions rather than standardized scales, which may have affected the accuracy of assessment. We considered building a sum score or using principal component analysis to combine the single PPF items. However, we decided against this approach because happiness and social support are distinct dimensions. Combining them into a single score could hide important details and lead to less meaningful conclusions. Another limitation is the high percentage of missing data for some PPFs, particularly for happiness and satisfaction with life and health (Supplementary material online, *Table S1*). Although XGBoost can handle missing data, we cannot entirely rule out that non-random missing patterns may have introduced bias into the prediction models. A further potential limitation is the possibility of a selection bias arising from the discrepancy between the overall sample size (475 175) and the smaller number of observations across various variables such as general happiness (162 972). Finally, we adjusted our estimations using an office-based version of the FRS not including lipid parameters, thereby not adjusting for dyslipidaemia.

Our results could be tentatively generalized to UK citizens, because participants were drawn from a large population-based UK cohort. However, it should be noted that the UKB sample is not fully representative of the general population of UK residents.^38^ According to the SCORE2 risk regions based on standardized cardiovascular disease mortality rates,^39^ results could be generalized to other low-CAD-risk countries, such as Norway, Denmark, The Netherlands, Belgium, France, Switzerland, and Spain.

## Implications

Our findings confirm the associations of happiness, satisfaction with health and life, and social support with reduced risk of AMI and CIHD. These findings underscore the recommendations of the 2021 ESC Guidelines on cardiovascular disease prevention^40^ that state that PPFs may act as CAD-risk modifiers, especially if the individual’s risk is close to a decision threshold. If conventional cardiovascular risk factors show an intermediate cardiovascular risk profile, PPFs such as happiness, satisfaction with health and life, and social support could modify risk prediction towards a low-risk category. Notably, the ESC Guidelines also mention other potential risk modifiers such as ethnicity, frailty, coronary calcification, genetics, and biomarkers (blood, urine, body composition).

Future research should focus on more comprehensive and in-depth assessments of PPFs such as well-being, purpose in life, optimism, and spirituality, favouring standardized scales, and questionnaires. Further, future studies comprising a larger number of AMIs/CIHDs are warranted to scrutinize whether associations between PPFs and AMI/CIHD are present during specific time windows following assessment or during the whole observation period. In addition, future research should explore more severe endpoints, such as CAD mortality. Notably, this requires a longer observation period, so that estimates could be based on a larger sample of subjects with cardiac events. Subgroup analysis should be considered to identify subpopulations in which PPFs might play a particularly relevant role. This could inform intervention studies, clinical practice, and future guidelines.

## Conclusions

Our analyses from a large-scale cohort study confirm that PPFs are associated with a reduced risk of CAD, consistent with previous research findings. However, incorporating available PPFs into both conventional and ML-based CAD‐prediction models did not improve prediction accuracy beyond that achieved with the FRS. Future research should focus on a more comprehensive and nuanced assessment of PPFs to better understand their potential predictive and protective value in preventive cardiology.

## Supplementary Material

zwae237_Supplementary_Data

## Data Availability

We requested and retrieved the data from the UKB in accordance with their guidelines and policies, which do not allow data transfer to third parties. Those interested in studying the UKB data can find information on how to apply and access the data on the UKB website (https://www.ukbiobank.ac.uk/enable-your-research).
