# Supplementary material for: Do positive psychosocial factors contribute to the prediction of coronary artery disease? A UK Biobank–based machine learning approach
Source: Eur J Prev Cardiol. 2024 Jul 26;32(6):443–52. doi: 10.1093/eurjpc/zwae237 (PMC12011491; doi:10.1093/eurjpc/zwae237)
Supplement: zwae237_Supplementary_Data [file zwae237_supplementary_data.docx]

# Supplementary Material

#### ***Table S1. Positive Psychosocial Factors***

|  | **Field ID** | **Variable** | ***n*** | **Proportion**^a^ |
| --- | --- | --- | --- | --- |
| **General Happiness** | | |  |  |
|  | 4526 | In general, how happy are you? | | |
|  |  | Extremely unhappy | 370 | 0.1% |
|  |  | Very unhappy | 1,161 | 0.2% |
|  |  | Moderately unhappy | 6,043 | 1.3% |
|  |  | Moderately happy | 83,741 | 18% |
|  |  | Very happy | 61,928 | 13% |
|  |  | Extremely happy | 8,714 | 1.8% |
|  |  | Do not know | 867 | 0.2% |
|  |  | Prefer not to answer | 414 | 0.1% |
|  |  | NA | 311,937 | 66% |
| **Satisfaction with Health** | | |  |  |
|  | 4548 | In general, how satisfied are you with your health? | | |
|  |  | Extremely unhappy | 1,692 | 0.4% |
|  |  | Very unhappy | 4,181 | 0.9% |
|  |  | Moderately unhappy | 15,143 | 3.2% |
|  |  | Moderately happy | 79,748 | 17% |
|  |  | Very happy | 52,979 | 11% |
|  |  | Extremely happy | 8,271 | 1.7% |
|  |  | Do not know | 876 | 0.2% |
|  |  | Prefer not to answer | 348 | 0.1% |
|  |  | NA | 311,937 | 66% |
| **Satisfaction with Life** | | |  |  |
|  | ​​4559 | In general, how satisfied are you with your family relationships? | | |
|  |  | Extremely unhappy | 992 | 0.2% |
|  |  | Very unhappy | 2,231 | 0.5% |
|  |  | Moderately unhappy | 7,406 | 1.6% |
|  |  | Moderately happy | 46,083 | 9.7% |
|  |  | Very happy | 72,596 | 15% |
|  |  | Extremely happy | 31,865 | 6.7% |
|  |  | Do not know | 1,155 | 0.2% |
|  |  | Prefer not to answer | 910 | 0.2% |
|  |  | NA | 311,937 | 66% |
|  | 4570 | In general, how satisfied are you with your friendships? | | |
|  |  | Extremely unhappy | 319 | 0.1% |
|  |  | Very unhappy | 782 | 0.2% |
|  |  | Moderately unhappy | 4,235 | 0.9% |
|  |  | Moderately happy | 51,252 | 11% |
|  |  | Very happy | 83,270 | 18% |
|  |  | Extremely happy | 20,911 | 4.4% |
|  |  | Do not know | 1,800 | 0.4% |
|  |  | Prefer not to answer | 669 | 0.1% |
|  |  | NA | 311,937 | 66% |
|  | 4581 | In general, how satisfied are you with your financial situation? | | |
|  |  | Extremely unhappy | 3,104 | 0.7% |
|  |  | Very unhappy | 5,504 | 1.2% |
|  |  | Moderately unhappy | 14,696 | 3.1% |
|  |  | Moderately happy | 74,283 | 16% |
|  |  | Very happy | 52,119 | 11% |
|  |  | Extremely happy | 11,811 | 2.5% |
|  |  | Do not know | 820 | 0.2% |
|  |  | Prefer not to answer | 901 | 0.2% |
|  |  | NA | 311,937 | 66% |
|  | 4537 | In general, how satisfied are you with the work that you do? | | |
|  |  | Extremely unhappy | 969 | 0.2% |
|  |  | Very unhappy | 2,145 | 0.5% |
|  |  | Moderately unhappy | 8,306 | 1.7% |
|  |  | Moderately happy | 53,166 | 11% |
|  |  | Very happy | 41,356 | 8.7% |
|  |  | Extremely happy | 9,612 | 2.0% |
|  |  | I am not employed | 46,275 | 9.7% |
|  |  | Do not know | 739 | 0.2% |
|  |  | Prefer not to answer | 670 | 0.1% |
|  |  | NA | 311,937 | 66% |
| **Social Support** | | |  |  |
|  | 1031 | How often do you visit friends or family or have them visit you? | | |
|  |  | Never or almost never | 6,769 | 1.4% |
|  |  | Once every few months | 31,474 | 6.6% |
|  |  | About once a month | 63,429 | 13% |
|  |  | About once a week | 167,726 | 35% |
|  |  | 2-4 times a week | 143,480 | 30% |
|  |  | Almost daily | 53,727 | 11% |
|  |  | No friends/family outside household | 1,167 | 0.2% |
|  |  | Do not know | 1,587 | 0.3% |
|  |  | Prefer not to answer | 1,447 | 0.3% |
|  |  | NA | 4,369 | 0.9% |
|  | 6160 | Leisure or social activities^b^ | | |
|  |  | 0 | 143,090 | 30% |
|  |  | 1 | 329,454 | 69% |
|  |  | NA | 2,631 | 0.6% |
|  | 2110 | How often are you able to confide in someone close to you? | | |
|  |  | Never or almost never | 66,142 | 14% |
|  |  | Once every few months | 25,785 | 5.4% |
|  |  | About once a month | 24,551 | 5.2% |
|  |  | About once a week | 50,836 | 11% |
|  |  | 2-4 times a week | 44,832 | 9.4% |
|  |  | Almost daily | 245,431 | 52% |
|  |  | Do not know | 13,819 | 2.9% |
|  |  | Prefer not to answer | 2,925 | 0.6% |
|  |  | NA | 854 | 0.2% |

NA, not available.

^a^ Percentages do not add up to one because of rounding.

^b^ We converted variable 6160 into a binary variable with values 0 if the participant answered *none of the above*, NA if there were no data available or participant answered *prefer not to answer* (1,803), and 1 if participant answered any of *Sports club or gym*, *Pub or social club*, *Religious group*, *Adult education class*, or *Other group activity*.

#### ***Table S2. UK Biobank Variables Used to Calculate the Framingham Risk Score***

| **Framingham score variable** | **UKB field ID** | **Processing steps** |
| --- | --- | --- |
| Sex | 31 | None |
| Age | 21022 | None |
| BMI | 21001 | None |
| Systolic blood pressure | 4080 | Mean of the two blood pressure measurements taken |
| Treatment of systolic blood pressure | 6177 (value 2 only) | Converted into a binary field for participants treated for systolic blood pressure |
|  | 6153 (value 2 only) |  |
|  | 20003 (curated list of medications^a^) |  |
| Current smoker | 20116 | Converted into a binary field for participants who answered they were current smokers (value 2) |
| Diabetes | 130706  130708  130710  130712  130714 | Combined all columns into one as a binary variable for diabetes status |
| BMI, body mass index; UKB, UK Biobank.  ^a^ 1140866144, 1140866330, 1141146124, 1141146126, 1141146128, 1140860308, 1140864202, 1140866146, 1140851364, 1140860332, 1140860404, 1140860422, 1140860562, 1140860738, 1140860764, 1140860790, 1140864950, 1140864952, 1140866138, 1140866162, 1140926778, 1141151016, 1141172682, 1141187788, 1141201038, 1141188636, 1140851362, 1140851660, 1140866164, 1141180592, 1140866078, 1140866092, 1140866094, 1140866096, 1140866102, 1140866104, 1140866108, 1140866110, 114115299 | | |

#### ***Table S3. Versions of the R (v4.1.2) Libraries and Packages Used in the Study***

| **Library** | **Version** |
| --- | --- |
| tidyverse | 1.3.1 |
| tidymodels | 0.2.0 |
| finetune | 0.2.0 |
| pROC | 1.18.0 |
| xgboost | 1.6.0.1 |
| vip | 0.3.2 |
| gtsummary | 1.6.1 |

#### ***Table S4. Crude Associations of Positive Psychosocial Factors with Coronary Artery Disease including P-for-Trend Analyses***

| Variable | | | AMI | | |  | CIHD | | |
| --- | --- | --- | --- | --- | --- | --- | --- | --- | --- |
|  |  |  | OR | 95% CI | p-value |  | OR | 95% CI | p-value |
| General Happiness | | |  |  |  |  |  |  |  |
|  | In General How Happy Are You (N = 163,238) | | | |  |  |  |  |  |
|  |  | Very or extremely unhappy | Reference category | | |  | Reference category | | |
|  |  | Moderately unhappy | 0.57 | 0.41, 0.80 | <0.001 |  | 0.69 | 0.57, 0.84 | <0.001 |
|  |  | Moderately happy | 0.56 | 0.43, 0.75 | <0.001 |  | 0.61 | 0.52, 0.73 | <0.001 |
|  |  | Very happy | 0.56 | 0.43, 0.75 | <0.001 |  | 0.59 | 0.50, 0.70 | <0.001 |
|  |  | Extremely happy | 0.66 | 0.49, 0.91 | 0.008 |  | 0.64 | 0.53, 0.77 | <0.001 |
|  |  | Do not know | 0.74 | 0.44, 1.21 | 0.2 |  | 0.81 | 0.60, 1.09 | 0.2 |
|  |  | Prefer not to answer | 0.49 | 0.20, 1.02 | 0.079 |  | 0.86 | 0.57, 1.24 | 0.4 |
|  |  | p for trend | 0.76 | 0.62, 0.94 | 0.01 |  | 0.71 | 0.63, 0.81 | <0.001 |
| Satisfaction with Health | | |  |  |  |  |  |  |  |
|  | In General How Satisfied Are You With Your Health (N = 163,238) | | | | |  |  |  |  |
|  |  | Extremely unhappy | Reference category | | |  | Reference category | | |
|  |  | Very unhappy | 1.05 | 0.76, 1.47 | 0.8 |  | 0.88 | 0.74, 1.05 | 0.15 |
|  |  | Moderately unhappy | 0.79 | 0.59, 1.08 | 0.13 |  | 0.63 | 0.54, 0.74 | <0.001 |
|  |  | Moderately happy | 0.7 | 0.53, 0.94 | 0.014 |  | 0.48 | 0.42, 0.56 | <0.001 |
|  |  | Very happy | 0.53 | 0.40, 0.72 | <0.001 |  | 0.32 | 0.28, 0.37 | <0.001 |
|  |  | Extremely happy | 0.52 | 0.38, 0.74 | <0.001 |  | 0.31 | 0.26, 0.37 | <0.001 |
|  |  | Do not know | 0.96 | 0.58, 1.55 | 0.9 |  | 0.57 | 0.43, 0.76 | <0.001 |
|  |  | Prefer not to answer | 0.77 | 0.34, 1.55 | 0.5 |  | 0.69 | 0.46, 1.00 | 0.061 |
|  |  | p for trend | 0.53 | 0.43, 0.65 | <0.001 |  | 0.33 | 0.30, 0.37 | <0.001 |
| Satisfaction with Life | | |  |  |  |  |  |  |  |
|  | Family Relationship Satisfaction (N = 163,238) | | |  |  |  |  |  |  |
|  |  | Very or extremely unhappy | Reference category | | |  | Reference category | | |
|  |  | Moderately unhappy | 0.62 | 0.47, 0.81 | <0.001 |  | 0.74 | 0.63, 0.87 | <0.001 |
|  |  | Moderately happy | 0.67 | 0.54, 0.84 | <0.001 |  | 0.72 | 0.63, 0.82 | <0.001 |
|  |  | Very happy | 0.69 | 0.56, 0.86 | <0.001 |  | 0.7 | 0.62, 0.80 | <0.001 |
|  |  | Extremely happy | 0.7 | 0.56, 0.88 | 0.001 |  | 0.75 | 0.66, 0.86 | <0.001 |
|  |  | Do not know | 0.82 | 0.52, 1.25 | 0.4 |  | 1.13 | 0.89, 1.42 | 0.3 |
|  |  | Prefer not to answer | 1.05 | 0.67, 1.60 | 0.8 |  | 1.14 | 0.88, 1.46 | 0.3 |
|  |  | p for trend | 0.82 | 0.71, 0.96 | 0.011 |  | 0.82 | 0.75, 0.90 | <0.001 |
|  | Friendships Satisfaction (N = 163,238) | |  |  |  |  |  |  |  |
|  |  | Very or extremely unhappy | Reference category | | |  | Reference category | | |
|  |  | Moderately unhappy | 0.64 | 0.42, 0.99 | 0.038 |  | 0.55 | 0.44, 0.70 | <0.001 |
|  |  | Moderately happy | 0.69 | 0.49, 1.02 | 0.049 |  | 0.61 | 0.50, 0.75 | <0.001 |
|  |  | Very happy | 0.69 | 0.49, 1.01 | 0.041 |  | 0.58 | 0.48, 0.71 | <0.001 |
|  |  | Extremely happy | 0.68 | 0.48, 1.00 | 0.04 |  | 0.57 | 0.46, 0.70 | <0.001 |
|  |  | Do not know | 0.97 | 0.62, 1.54 | 0.9 |  | 0.78 | 0.60, 1.02 | 0.066 |
|  |  | Prefer not to answer | 0.52 | 0.24, 1.04 | 0.078 |  | 0.79 | 0.56, 1.11 | 0.2 |
|  |  | p for trend | 0.8 | 0.63, 1.03 | 0.075 |  | 0.71 | 0.62, 0.82 | <0.001 |
|  | Financial Situation Satisfaction (N = 163,238) | | |  |  |  |  |  |  |
|  |  | Extremely unhappy | Reference category | | |  | Reference category | | |
|  |  | Very unhappy | 0.82 | 0.62, 1.09 | 0.2 |  | 0.81 | 0.69, 0.95 | 0.01 |
|  |  | Moderately unhappy | 0.78 | 0.62, 1.01 | 0.052 |  | 0.73 | 0.64, 0.84 | <0.001 |
|  |  | Moderately happy | 0.76 | 0.61, 0.96 | 0.015 |  | 0.71 | 0.62, 0.80 | <0.001 |
|  |  | Very happy | 0.65 | 0.52, 0.82 | <0.001 |  | 0.64 | 0.56, 0.73 | <0.001 |
|  |  | Extremely happy | 0.69 | 0.54, 0.90 | 0.005 |  | 0.64 | 0.56, 0.75 | <0.001 |
|  |  | Do not know | 0.96 | 0.57, 1.52 | 0.9 |  | 0.98 | 0.74, 1.28 | 0.9 |
|  |  | Prefer not to answer | 0.87 | 0.52, 1.38 | 0.6 |  | 0.87 | 0.66, 1.14 | 0.3 |
|  |  | p for trend | 0.74 | 0.62, 0.87 | <0.001 |  | 0.7 | 0.64, 0.77 | <0.001 |
|  | Work Or Job Satisfaction (N = 163,238) | | |  |  |  |  |  |  |
|  |  | Very or extremely unhappy | Reference category | | |  | Reference category | | |
|  |  | Moderately unhappy | 0.87 | 0.65, 1.20 | 0.4 |  | 0.88 | 0.74, 1.06 | 0.2 |
|  |  | Moderately happy | 0.88 | 0.68, 1.16 | 0.3 |  | 0.91 | 0.78, 1.07 | 0.2 |
|  |  | Very happy | 0.92 | 0.71, 1.22 | 0.5 |  | 1 | 0.86, 1.18 | >0.9 |
|  |  | Extremely happy | 1.12 | 0.84, 1.52 | 0.4 |  | 1.05 | 0.88, 1.25 | 0.6 |
|  |  | I am not employed | 1.34 | 1.04, 1.76 | 0.031 |  | 1.58 | 1.35, 1.85 | <0.001 |
|  |  | Do not know | 1.22 | 0.69, 2.06 | 0.5 |  | 1.4 | 1.02, 1.90 | 0.034 |
|  |  | Prefer not to answer | 1.02 | 0.54, 1.82 | >0.9 |  | 1.5 | 1.08, 2.05 | 0.012 |
|  |  | p for trend | 1.09 | 0.90, 1.33 | 0.4 |  | 1.07 | 0.96, 1.21 | 0.2 |
| Social Support | | |  |  |  |  |  |  |  |
|  | Frequency Of Friend Or Family Visits (N = 470,806) | | | |  |  |  |  |  |
|  |  | Never or almost never | Reference category | | |  | Reference category | | |
|  |  | Once every few months | 0.67 | 0.58, 0.78 | <0.001 |  | 0.68 | 0.62, 0.74 | <0.001 |
|  |  | About once a month | 0.6 | 0.52, 0.69 | <0.001 |  | 0.6 | 0.55, 0.65 | <0.001 |
|  |  | About once a week | 0.64 | 0.56, 0.74 | <0.001 |  | 0.63 | 0.58, 0.69 | <0.001 |
|  |  | 2-4 times a week | 0.67 | 0.58, 0.77 | <0.001 |  | 0.66 | 0.61, 0.72 | <0.001 |
|  |  | Almost daily | 0.71 | 0.62, 0.83 | <0.001 |  | 0.77 | 0.70, 0.84 | <0.001 |
|  |  | No friends/family outside household | 0.97 | 0.68, 1.36 | 0.9 |  | 1.24 | 1.01, 1.51 | 0.034 |
|  |  | Do not know | 0.98 | 0.71, 1.32 | >0.9 |  | 0.95 | 0.78, 1.15 | 0.6 |
|  |  | Prefer not to answer | 0.76 | 0.53, 1.07 | 0.13 |  | 0.78 | 0.63, 0.97 | 0.025 |
|  |  | p for trend | 0.82 | 0.75, 0.90 | <0.001 |  | 0.85 | 0.81, 0.90 | <0.001 |
|  | Leisure Or Social Activities (N = 472,544) | | 0.92 | 0.88, 0.96 | <0.001 |  | 0.93 | 0.91, 0.96 | <0.001 |
|  | Able To Confide (N = 474,321) | |  |  |  |  |  |  |  |
|  |  | Never or almost never | Reference category | | |  | Reference category | | |
|  |  | Once every few months | 0.74 | 0.67, 0.82 | <0.001 |  | 0.79 | 0.75, 0.84 | <0.001 |
|  |  | About once a month | 0.72 | 0.65, 0.80 | <0.001 |  | 0.73 | 0.68, 0.77 | <0.001 |
|  |  | About once a week | 0.7 | 0.65, 0.76 | <0.001 |  | 0.71 | 0.68, 0.75 | <0.001 |
|  |  | 2-4 times a week | 0.67 | 0.61, 0.72 | <0.001 |  | 0.66 | 0.62, 0.69 | <0.001 |
|  |  | Almost daily | 0.75 | 0.71, 0.79 | <0.001 |  | 0.74 | 0.72, 0.77 | <0.001 |
|  |  | Do not know | 0.93 | 0.83, 1.04 | 0.2 |  | 0.93 | 0.87, 1.00 | 0.042 |
|  |  | Prefer not to answer | 0.93 | 0.73, 1.16 | 0.5 |  | 0.97 | 0.84, 1.11 | 0.7 |
|  |  | p for trend | 0.8 | 0.76, 0.85 | <0.001 |  | 0.78 | 0.76, 0.81 | <0.001 |

AMI, acute myocardial infarction; CI, Confidence Interval; CIHD, chronic ischaemic heart disease; *OR*, Odds Ratio.

#### ***Table S5. TRIPOD Guidelines Report***

| **Section/Topic** | **Item** | **Checklist Item** | **Page** |
| --- | --- | --- | --- |
| **Title and abstract** | | | |
| Title | 1 | Identify the study as developing and/or validating a multivariable prediction model, the target population, and the outcome to be predicted. | 0 |
| Abstract | 2 | Provide a summary of objectives, study design, setting, participants, sample size, predictors, outcome, statistical analysis, results, and conclusions. | 1 |
| **Introduction** | | | |
| Background and objectives | 3a | Explain the medical context (including whether diagnostic or prognostic) and rationale for developing or validating the multivariable prediction model, including references to existing models. | 4 |
|  | 3b | Specify the objectives, including whether the study describes the development or validation of the model or both. | 4 |
| **Methods** | | | |
| Source of data | 4a | Describe the study design or source of data (e.g., randomized trial, cohort, or registry data), separately for the development and validation data sets, if applicable. | 5 |
|  | 4b | Specify the key study dates, including start of accrual; end of accrual; and, if applicable, end of follow-up. | 5 |
| Participants | 5a | Specify key elements of the study setting (e.g., primary care, secondary care, general population) including number and location of centres. | 5 |
|  | 5b | Describe eligibility criteria for participants. | 5 |
|  | 5c | Give details of treatments received, if relevant. | NA |
| Outcome | 6a | Clearly define the outcome that is predicted by the prediction model, including how and when assessed. | 5 |
|  | 6b | Report any actions to blind assessment of the outcome to be predicted. | NA |
| Predictors | 7a | Clearly define all predictors used in developing or validating the multivariable prediction model, including how and when they were measured. | 5 |
|  | 7b | Report any actions to blind assessment of predictors for the outcome and other predictors. | NA |
| Sample size | 8 | Explain how the study size was arrived at. | NA |
| Missing data | 9 | Describe how missing data were handled (e.g., complete-case analysis, single imputation, multiple imputation) with details of any imputation method. | 6 |
| Statistical analysis methods | 10a | Describe how predictors were handled in the analyses. | 6 |
|  | 10b | Specify type of model, all model-building procedures (including any predictor selection), and method for internal validation. | 6 |
|  | 10d | Specify all measures used to assess model performance and, if relevant, to compare multiple models. | 5 |
| Risk groups | 11 | Provide details on how risk groups were created, if done. | NA |
| **Results** | | | |
| Participants | 13a | Describe the flow of participants through the study, including the number of participants with and without the outcome and, if applicable, a summary of the follow-up time. A diagram may be helpful. | 7 |
|  | 13b | Describe the characteristics of the participants (basic demographics, clinical features, available predictors), including the number of participants with missing data for predictors and outcome. | 7 |
| Model development | 14a | Specify the number of participants and outcome events in each analysis. | 7 |
|  | 14b | If done, report the unadjusted association between each candidate predictor and outcome. |  |
| Model specification | 15a | Present the full prediction model to allow predictions for individuals (i.e., all regression coefficients, and model intercept or baseline survival at a given time point). | 7, 19 |
|  | 15b | Explain how to use the prediction model. | 5 |
| Model performance | 16 | Report performance measures (with CIs) for the prediction model. | 7-8 |
| **Discussion** | | | |
| Limitations | 18 | Discuss any limitations of the study (such as nonrepresentative sample, few events per predictor, missing data). | 10 |
| Interpretation | 19b | Give an overall interpretation of the results, considering objectives, limitations, and results from similar studies, and other relevant evidence. | 9 |
| Implications | 20 | Discuss the potential clinical use of the model and implications for future research. | 11 |
| **Other information** | | | |
| Supplementary information | 21 | Provide information about the availability of supplementary resources, such as study protocol, Web calculator, and data sets. | 11 |
| Funding | 22 | Give the source of funding and the role of the funders for the present study. | 12 |
